# *Anopheles arabiensis* in Sudan: a noticeable tolerance to urban polluted larval habitats associated with resistance to Temephos

**DOI:** 10.1186/s12936-018-2350-1

**Published:** 2018-05-18

**Authors:** Rasha S. Azrag, Babiker H. Mohammed

**Affiliations:** 1 Department of Zoology, Faculty of Science, University of Khartoum, Khartoum, Sudan; 2 Ministry of Health, Khartoum State, Malaria Control Department, Vector Surviellance Unit, Khartoum, Sudan

**Keywords:** Pollution, Larval habitats, *Anopheles arabiensis*, Sudan

## Abstract

**Background:**

It has been documented that unplanned urbanization leads to the exposure of members of the *Anopheles* vectors to a range of water pollution in urban settings. Many surveys from African and Asian countries reported the presence of *Anopheles* larvae in polluted urban habitats. The present study documents an obvious tolerance of the melanic and normal forms of *Anopheles arabiensis* to urban polluted larval habitats accompanied by resistance to Temephos larvicide.

**Methods:**

A cross-sectional survey was carried out to inspect apparently polluted *An. arabiensis* larval habitats during the hot dry season of 2015. Larval specimens were collected from only apparently polluted habitats after visual inspection from 5 localities in Khartoum State. After morphological and molecular identification of random samples of larvae the magnitude of water pollution was determined using nine abiotic factors. The susceptibility status of *An. arabiensis* larval forms from normal and polluted habitats to Temephos was tested using the WHO standard diagnostic concentration doses.

**Results:**

Morphological and PCR analysis of anopheline larvae revealed the presence of *An. arabiensis*, a member of the *Anopheles gambiae* complex. Seven out of 9 physiochemical parameters showed higher concentrations in polluted larval habitats in comparison to control site. *Anopheles arabiensis* larvae were found in water bodies characterized by high mean of conductivity (1857.8 ± 443.3 uS/cm), turbidity (189.4 ± 69.1 NTU) and nitrate (19.7 ± 16.7 mg/l). The range of mortality rates of *An. arabiensis* larvae collected from polluted habitats in comparison to *An. arabiensis* larvae collected from non-polluted habitats was 6.7–64% (LD_50_ = 1.682) and 67.6–96% (LD_50_ = 0.806), respectively.

**Conclusions:**

The present study reveals that minor populations of *An. arabiensis* larval forms are adapted to breed in polluted urban habitats, which further influenced susceptibility to Temephos, especially for the melanic larval forms. This could have further implications on the biology of the malaria vector and on the transmission and epidemiology of urban malaria in Sudan.

## Background

During the past 10 years many studies have stated that *Anopheles* species were adapted to polluted habitats in urban settings, the majority from African countries. *Anopheles gambiae* sensu stricto (s.s.) in urban settings in Nigeria are adapting to a wide range of water pollution [1].

In urban cities in Ghana, *An. gambiae* was found in polluted aquatic habitats and this, coupled with occurrence of insecticide resistance, is alarming [2]. In Yaoundé, Cameroon, the presence of *An. gambiae* in organically polluted sites has been confirmed [3]. Other studies such as in Pakistan and Sri Lanka indicated the adaptation of *Anopheles gambiae* sensu lato (s.l.) and *Anopheles culicifacies* to organically polluted water habitats [4, 5] and reported that rapid, unplanned urbanization is considered to favour adaptation of anophelines to various xenobiotics and the expansion of their niche to polluted habitats. *Anopheles gambiae* complex, which includes the major vectors in Africa, has the capacity to exploit different kinds of habitat that are created either directly or indirectly by humans. This is evidenced by its wide geographical distribution and its occurrence in a variety of micro- and macro-environmental conditions throughout tropical Africa, and that larvae were found in habitats organically polluted by rotting vegetation, human faeces, or oil [2, 6, 7].

*Anopheles arabiensis*, a sibling species of the *An. gambiae* complex, is a predominant malaria vector all over Sudan, covering arid and semi-arid areas of the country. It is the only malaria vector encountered in Khartoum State [8], the capital of Sudan, which have long been Sudan’s primary industrial, commercial and manufacturing hubs with very poor infrastructure that has many environmental consequences [9]. The present study documents, the presence of *An. arabiensis* larvae in polluted habitats in urban Khartoum, central Sudan, which further influenced its susceptibility to insecticides.

## Methods

### Study area

**Fig. 1:**
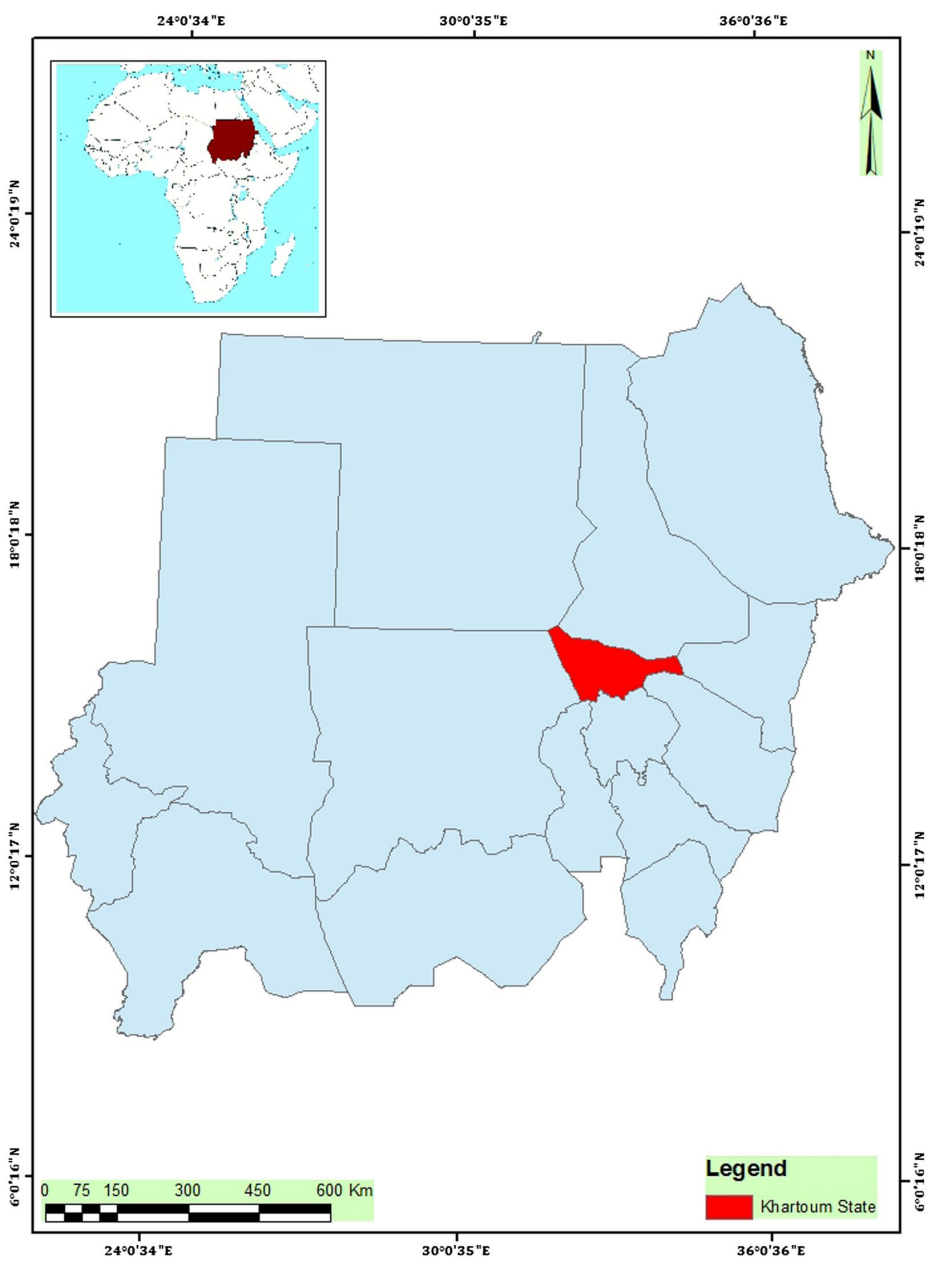
Geographic location of Khartoum state

This study was carried out in Khartoum in central Sudan, at the confluence of the White Nile and Blue Nile, (31.5–34°E and 15–16°N), over 250 km and a total area of 20,736 sq km (Fig. 1). According to the Sudan Meteorological Authority (SMA), the temperatures range between 25 and 40 °C during the months April to June and between 20 and 35 °C during July to October. Temperatures fall during the winter period between November and March to 15–25 °C.

Most of Khartoum State falls within the semi-desert climatic zone while the northern part falls within the desert climatic zone. The State is prevailed with a hot to very hot rainy season during the summer and warm to cold dry season during the winter. Rainfall ranges between 100 and 200 mm in northeastern parts, 200–300 mm in southern parts and 10,100 mm in the northwestern parts.

Khartoum State has a population of 5,271,321, according to the Census conducted in 2008 by the Central Bureau of Statistics [10]. Khartoum’s drainage and sanitation systems are in poor condition. Only 28% of Khartoum is connected to a sewage system, and most residents use pit latrines and other basic systems such as septic tanks and siphon wells.

### Larval surveillance

Two cross-sectional larval surveillance surveys were carried out in 5 localities in Khartoum State: Khartoum, East Nile, Jubal Aulia, Khartoum North, and Omdurman. The first survey was carried out during the hot dry season from February to April 2015 and was designed to study the possibility of *Anopheles* larvae to breed in polluted aquatic habitats. The second surveillance was conducted during the late rainy season from September to November 2015 to examine the susceptibility of *An. arabiensis* found in polluted water bodies to Temephos larvicide.

### Survey 1

Larvae specimens were collected from only apparently polluted habitats after visual inspections. According to [3, 6], polluted larval habitats of *An. gambiae* s.l. are: “semi-permanent water collections containing domestic waste or organic products in decomposition that could be invaded by moisture and/or algae”. The expectation of polluted larval habitats in urban areas came after: visual inspection (colour and odour); presence near drainage system; presence near industrial areas presence near effluent from local houses and presence near university campus and military camps.

A handheld global positioning system (GPS) was used to determine and record the precise grid co-ordinates of the positive larval habitats. Samples of *Anopheles* larvae present in suspected polluted habitats were collected using standard dipping methods and preserved in 80% ethanol in small glass bottles with full labels. Water samples from each site were collected concurrently to study physiochemical parameters and preserved in clean plastic bottles (600 ml).

### Survey 2

Mosquito larvae were collected from 4 localities in Khartoum State: Khartoum, Khartoum North, East Nile, and Jabal Awlia, to determine susceptibility of *An. arabiensis* larvae in polluted larval bodies to Temephos larvicide. Samples were collected from two types of habitats:

- Polluted *Anopheles* species habitats that represented the positive *Anopheles* larvae habitats identified during Survey 1.
- Non-polluted *Anopheles* species larval habitats in agricultural areas within urban Khartoum State.

### Collection, preservation and identification methods

*Anopheles* larvae were sampled using standard dipping method; from each breeding site, 10 dips were taken with a standard 300-ml dipper according to [11]. Larvae were preserved in 80% ethanol in glass bottles. In the laboratory, larvae were identified morphologically using [12] and molecularly using species-specific identification according to [13]. Melanic and normal forms were classified according to [14].

### Physicochemical analysis of water samples

Water samples were collected from each visually polluted larval habitat in 600-ml clean plastic bottles. To study the magnitude of water pollution at the breeding sites eight abiotic factors were determined, including colour, odour, pH, conductivity, total dissolved solids, turbidity, oil and nitrate. Water pollution associated with heavy metals was determined for three heavy metals (Cu, Pb, Fe), which were chosen as they were previously studied in *An. gambiae* s.s. polluted larval habitats [1]. Water samples were analysed in the Central Laboratory, Chemistry Department, Khartoum University. pH, conductivity and total dissolved solids (TDS) were analysed using pH 315i/SET, NO: 2A10-1012 and Cond 315i/SET, NO: 2C10-001, Germany, and turbidity was measured using Palintest: Turbimerer, PT 0900513197, UK. Heavy metals were determined using atomic absorption spectrophotometry at the Central Laboratory, Chemistry Department, Khartoum University. Physiochemical parameters from apparently polluted larval habitats were compared to physiochemical parameters of a control larval habitat (an irrigation canal at an agricultural area within Khartoum State).

### Bioassay tests

The susceptibility bioassays were performed according to [15]. Twenty-five late third to early fourth-instar larvae of *An. arabiensis* were laboratory assayed for sensitivity to Temephos larvicide using four diagnostic concentrations; 0.005, 0.025, 0.125, and 0.625 mg/l. The average temperature of the water was 25 °C. Each concentration was replicated three times. After a period of 24 h, mortality counts were performed. Control trials were performed under same conditions.

### Data analysis

Data were analysed using SPSS version 15.0. Descriptive statistics were used. The relationship between habitats variables and presence of anophelines in polluted water was tested using correlation. Only variables with P values < 0.05 were considered. The results of the insecticide susceptibility tests were analysed for dose/response relationship using regression probit analysis [16]. The 50% lethal concentration or LC_50_ value is commonly accepted as the basis for comparison in the investigation of relative toxicities among insecticides used.

## Results

### Identification of *Anopheles* larvae

A total of 377 larvae were collected during Survey 1. Random samples of larvae were reared in the laboratory and all larvae successfully completed their development and emerged as adults. All samples were identified as *An. gambiae* complex and all PCR products of the amplification of intergenic spacer region (IGS) of ribosomal DNA (rDNA) of larvae specimens showed the diagnostic fragments of 315 base pairs that identify *An. arabiensis*. Normal and melanic larval forms were identified from all types of larval habitats with a noticeable increase of the melanic in polluted larval habitats described below.

### Types of polluted larval habitats

**Fig. 2:**
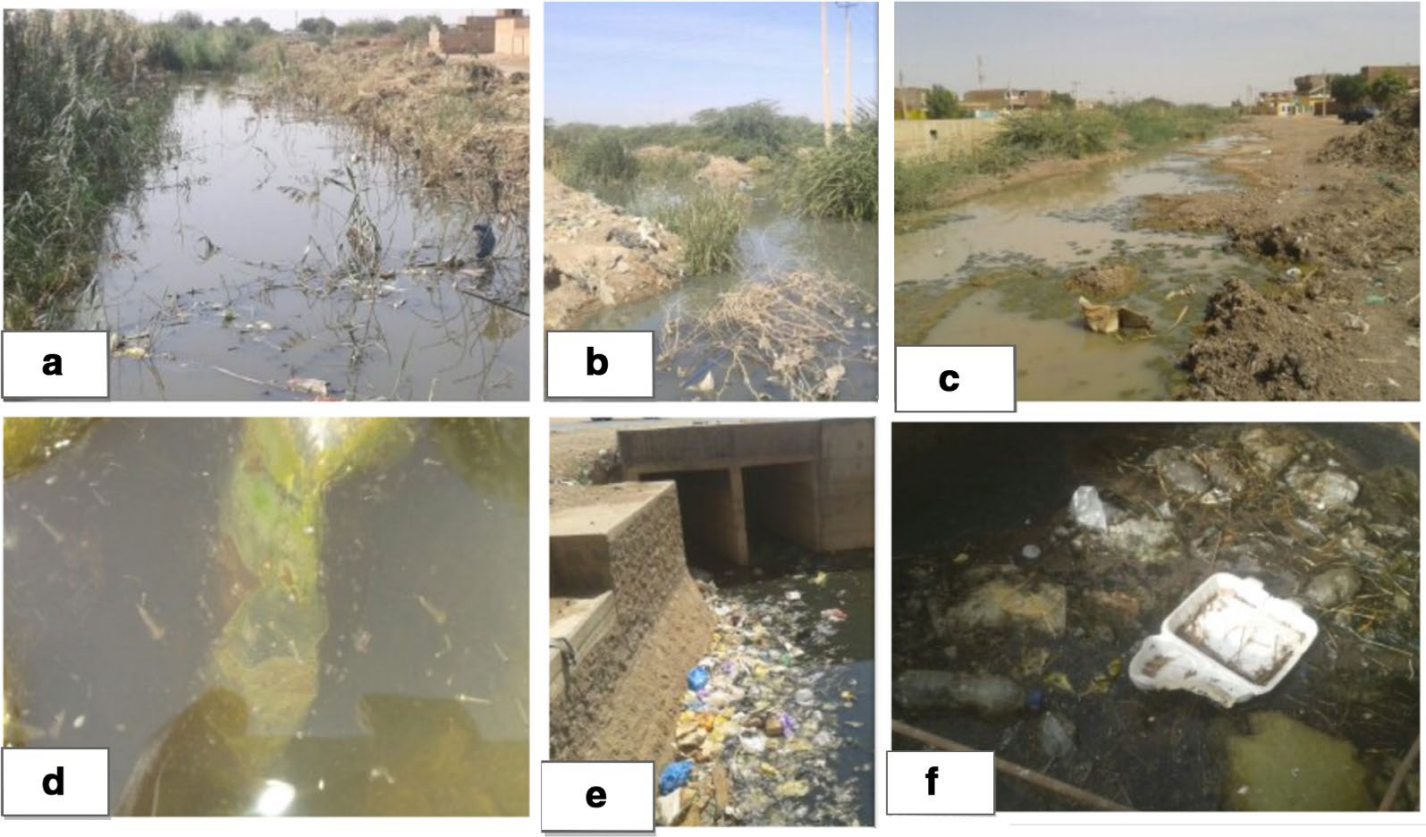
Types of polluted habitats in Khartoum State. **a** main canal of plant treatment; **b** junction of tributaries of main sewage canal; **c** pools created from leakage of main sewage canal; **d** polluted pond; **e** a culvert on a drainage canal; **f** manhole sewers

*Anopheles arabiensis* larvae were collected from 17 polluted larval habitats that were disproportionately divided between the 5 localities. Most prevalent types of polluted larval habitats were drains of concrete sewers (39%) followed by pools created from sewage canal and industrial effluent, both constituted 17% and manholes and drainage canal both constituted 11%. Types and nature of polluted larval habitats are shown in Fig. 2.

Khartoum North and East Nile localities both represented 56% of the total positive polluted larval habitats, followed by Omdurman 17%, Khartoum 16, and 11% Jabal Awlia.

### Larval density per type of habitat

**Fig. 3:**
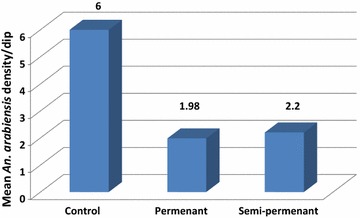
Density of *Anopheles arabiensis* in polluted larval habitats in comparison to control site during the hot dry season

Polluted larval habitats were classified into either semi-permanent (available for 3 months) and permanent habitats (available for more than 3 months) without any type of temporary habitats. Two types of polluted habitats were noticed, permanent and semi-permanent: 61% of polluted habitats were permanent and 39% were semi-permanent. Highest mean density/dip of *An. arabiensis* larvae was recorded in semi-permanent habitats (2.2/dip) in comparison to permanent habitats (1.98/dip) (Fig. 3).

### Physicochemical parameters of polluted larval habitat

Table 1 summarizes the polluted habitats of *An. arabiensis* in Khartoum State, their positions, types and physiochemical descriptions. The pH ranged from 6.8 to 8.0 in polluted habitats, which was 1.1-fold higher in comparison to control site. The highest pH was observed in East Nile in Eilafun camp (8.0) while the lowest one was recorded in Khartoum North in Elsababi (6.8). Khartoum North showed highest mean of conductivity in Khartoum North—Industrial Area (6650 uS/cm) and the lowest was observed in Khartoum North-Wad—ELsaaeh (313 uS/cm). The mean of turbidity (NTU) in polluted habitats was (189.4 ± 69.1), which was 52.6-fold in comparison to control site (3.6). East Nile -Hag Yousif (Shiglah) registered the highest NTU (1012) and the lowest was observed in East Nile—Eilafun military camp (2.9 NTU).

**Table 1:** Physicochemical properties of water samples collected from different urban polluted larval habitats of *Anopheles arabiensis* in Khartoum state

ID	Site	Type of larval habitats	Contamination type	Depth (cm)	Grass	Algae	Den/dip (TN)	Color	Odor	Abiotic variables						Heavy metals		
										pH	Cond (uS/cm)	TDS (mg/l)	Turbidity (NTU)	Oil (mg/l)	Nitrate (mg/l)	Cu (mg/l)	Pb (mg/l)	Fe (mg/l)
1	Gabal Awlia	Semi-permanent	Ponds from sewage water canal	15	Yes	Yes	2	Yellowish	Bad	7.63	1967	1376.9	134	0.0190	303	0.0086	0.1068	N.D.
2		Semi-permanent	Ponds from sewage water canal	30	Yes	Yes	1	Yellowish	Bad	7.84	2350	1645	209	0.10	9.357	0.0152	N.D.	2.0181
3	Khartoum	Semi-permanent	Pond from sewage water canal	40	Yes	Yes	0.9	Yellowish	Bad	7.65	1618	1132.6	483	0.21	7.686	0.0065	0.0138	0.1252
4		Permanent	Sewage water canal	70	No	Yes	0.3	Redish	Bad	7.45	6160	4312	231	0.07	11.660	0.0011	N.D.	N.D.
5		permanent	Sewage canal of industrial area	30	Yes	Yes	1.1	Clean	Bad	7.22	490	343	28.8	–	–	–	–	–
6	East Nile	Permanent	Accumulations of houses sewage water	25	Yes	No	1.5	Clean	Bad	7.30	519	363.3	4.66	–	–	–	–	–
7		Semi-permanent	Concrete water tank	15	No	No	3.8	Greenish	Bad	7.67	580	406	14.4	0.034	3.606	0.0119	N.D.	0.4151
8		Permanent	Sewage water manhol	20	No	Yes	1.4	Clean	Bad	8.05	3430	2401	2.99	0.44	7.008	N.D.	0.0345	0.9232
9		Permenant	Accumulations of houses sewage water	10	Yes	Yes	5.6	Blackish	Bad	6.93	1119	783.3	1012	0.0440	6.90	0.0140	0.1068	0.4487
10		Permanent	Irrigated water canal^a^	10	Yes	Yes	6	Clean	Normal	6.93	216	151.2	3.60	0.0090	4.30	0.0173	0.0667	N.D.
11	Khartoum North	Semi-permanent	Waste water canal	20	Yes	Yes	1.5	Clean	Bad	7.15	324	226.8	653	0.15	2.144	N.D.	N.D.	0.7908
12		Semi-permanent	Waste water from a factory	25	No	No	1.4	Redish	Bad	7.99	6650	4655	552	–	–	–	–	–
13		Semi-permanent	Accumulations of houses sewage water	25	Yes	Yes	1.6	Clean	Bad	7.16	313	219.1	8.44	–	–	–	–	–
14		Semi-permanent	Accumulations of houses sewage water	10	Yes	Yes	3.7	Clean	Bad	7.40	2440	1708	9.49	–	–	–	–	–
15		Semi-permanent	Accumulations of houses sewage water	20	No	Yes	1.6	Clean	Bad	6.81	782	547.4	26.9	–	–	–	–	–
16	Omdurman	Semi-permanent	Accumulations of houses sewage water	15	Yes	Yes	2	Yellowish	Bad	7.18	1655	1158.5	8.26	–	–	–	–	–
17		Semi-permanent	Accumulations of houses sewage water	60	Yes	Yes	0.6	Clean	Bad	7.18	830	581	22	–	–	–	–	–
18		Semi-permanent	Accumulations of houses sewage water	20	Yes	Yes	1.7	Clean	Bad	7.36	1998	1398.6	6.44	–	–	–	–	–

*N.D.* Not detected, – Not done

^a^Normal *An. arabiensis* larval habitat

The mean of oil in polluted habitats was (0.06 ± 0.02 mg/l) which was 6.7 times higher in comparison to control site (0.009 mg/l). However, very high concentration of oil was recorded in East Nile—Eilafun military camp (0.4 mg/l). The mean of nitrate in polluted habitats was (19.7 ± 16.7) which was 4.6 times higher in comparison to control site (4.3). The highest concentration of nitrate was observed in Jebel Awlia-Abu Adam (303 mg/l).

The mean of Fe (mg/l) in polluted larval habitats was (0.3 ± 0.1) in comparison to 0 Fe mg/l in control site. Jebel Awlia—Block (1 + 3) showed highest mean of Fe (2.0 mg/l). Regarding Cu (mg/l) in polluted habitats the mean was (0.004 ± 0.001) which was fivefold lower in comparison to control site and was not detected in Khartoum North-Kober and East Nile-Eilafun camp. Similarly, concentration of Pb in polluted habitats was (0.02 ± 0.01 mg/l) which was 3.5 times lower in comparison to control site. Table 2 shows types and geographical locations of polluted larval habitats.

**Table 2:** Types and geographical positions of positive polluted *An. arabiensis* larval habitats in Khartoum state

Locality	Site	GPS position	Type of larval habitats	Permanent/semi-permanent
East Nile	Elshiglah	15°38.547´N, 32°37.545´E	Drainage canal (Khor)	Permanent
	Eldoha	15°37.083´N, 32°35.873´E	Drains of concrete sewer	Permanent
	Eilafun Hospital	15°25.287´N, 32°43.848´E	Manhole	Semi-permanent
	Eilafun Camp	15°24.884´N, 32°43.775´E	Manhole	Permanent
	Selate (Control)	15°34.622´N, 32°39.908´E	Irrigated canal (Abueshreen)	Permanent (irrigated project)
Khartoum North	Kober	15°38.057´N, 32°32.855´E	Drains of concrete sewer	Permanent
	Indust-area	15°38.120´N, 32°33.075´E	Industrial effluents	Permanent
	Elsababi	15°37.981´N, 32°31.774´E	Drainage canal (Khor)	Semi-permanent
	Wadelsaeh	15°38.511´N, 32°30.797´E	Drains of concrete sewer	Permanent
	Hilatkhogali	15°37.855´N,32°30.944´E	Drains of concrete sewer	Permanent
Khartoum	Industrial area	15°35.215´N, 32°30.573´E	Industrial effluents	Semi-permanent
	Elrimalah	15°33.574´N, 32°30.361´E	Industrial effluents	Permanent
	Eldubaseen	15°30.483´N, 32°29.745´E	Pools of sewage canal	Semi-permanent
Omdurman	Baitelmal	15°38.976´N, 32°30.226´E	Drains of concrete sewer	Permanent
	Elhigrah	15°39.441´N, 32°29.905´E	Drains of concrete sewer	Semi-permanent
	Elmulazmeen	15°37.898´ N, 32°29.473´E	Drains of concrete sewer	Permanent
Jabel Awlia	Abu Adam	15°30.612´N, 32°29.937´E	Pools of sewage canal	Semi-permanent
	Block (1, 3)	15°30.486´N, 32°29.124´E	Pools of sewage canal	Semi-permanent

### Physicochemical characteristics of polluted larval habitats per locality

When data of physiochemical parameters of polluted habitats were analysed according to localities, the average larval density per dip per locality was higher in East Nile (3.1 larvae/dip) followed by Khartoum (2 larvae/dip) and Jabal Awlia (1.5 larvae/dip). The pH readings of the water samples across all localities were higher than the readings of control site. The conductivity recorded across all localities was sixfold higher than the control site. However, Khartoum, Jabal Awlia and Khartoum North were more than ninefold higher than control site (2756, 2158.5, 2101.8 and 216 uS/cm, respectively) (Table 3).

**Table 3:** Physicochemical parameters per locality

Site	Density per dip	PH	Conductivity (uS/cm)	TDS (Mg/l)	Turbidity (NTU)	Nitrate (mg/l)	Oil (mg/l)	Cu (mg/l)	Pb (mg/l)	Fe (mg/l)
East Nile	3.1	7.5	1412	988.4	258.5	5.84	0.173	0.0086	0.0471	0.595
Jabal Awlia	1.5	7.7	2158.5	1511	171.5	156.2	0.0595	0.0119	0.0534	1.009
Khartoum North	0.8	7.3	2101.8	1471.3	250	2.14	0.15	–	–	0.7908
Khartoum	2	7.4	2756	1929.2	247.6	9.67	0.14	0.0038	0.0069	0.0626
Omdurman	1.4	7.2	1494.3	1046.1	12.2	–	–	–	–	–
Control	6	6.9	216	151.2	3.6	4.3	0.009	0.0173	0.0667	ND

*ND* not done

Jabal Awlia was characterized as having a highest mean of nitrate (156.2 NTU) and was 36-fold higher than the value of nitrate recorded in control site (4.3 NTU). Jabal Awlia recorded highest level of Fe (1.009 mg/l) in comparison to other localities.

### Temephos bioassay

Three different bioassay tests were done, each with three replicates along with control tests. The first and second tests were conducted to evaluate susceptibility status of the melanic (M) and normal (N) forms collected from non-polluted habitats with a total of 358 and 374 larvae, respectively. The third test was done to evaluate susceptibility status of *An. arabiensis* larvae found in polluted habitats and included 373 larvae that were collected from the polluted sites identified in Survey 1.

Table 4 shows 100% mortality rates for *An. arabiensis* M, when exposed to 0.125 and 0.625 mg/l concentrations of Temephos. However, lower concentrations showed that lower mortality rates ranged between 87.3 and 16.2%. *Anopheles arabiensis* N larvae showed similar mortality rates for 0.125, 0.625 and 0.025 mg/l concentrations of Temephos, with mortality rates ranged between 100 and 96%. However, lower mortality rates (67.6%) were reported for 0.005 mg/l concentration. One-hundred percent mortality rates were reported for *An. arabiensis* larvae collected from polluted habitats for 0.125 and 0.625 mg/l concentrations of Temephos. Lower mortality rates ranging between 64 and 6.7% were reported for 0.025 and 0.005 mg/l concentrations, respectively.

**Table 4:** Mortality rates of *An. arabiensis* forms exposed to different concentrations of Temephos insecticide

Concentrations (mg/l)	*An. arabiensis* melanic form (M)			*An. arabiensis* normal form (N)			*An. arabiensis* from polluted sites		
	Number exposed	Number dead	Mortality %	Number exposed	Number dead	Mortality %	Number exposed	Number dead	Mortality %
0.005	74	12	16.2	74	50	67.6	75	5	6.7
0.025	71	62	87.3	75	72	96	75	48	64
0.125	68	68	100	75	75	100	74	74	100
0.625	70	70	100	75	75	100	75	75	100

### Calculations of LD_50_ and LD_95_

Higher LD_50_ to Temephos was reported with *An. arabiensis* M (1.369 mg/l) in comparison to *An. arabiensis* N (0.806 mg/l). Regarding *An. arabiensis* collected from polluted habitats LD_50_ was 1.682 mg/l and LD_95_ was 2.785 mg, which indicates resistance to Temephos (Table 5).

**Table 5:** Lethal concentration 50% (LD_50_) and 95% (LD_95_) of *An. arabiensis* larval forms exposed to WHO discriminative doses of Temephos insecticides

Species	95% confidence limits LD_50_ (lower–upper)	95% confidence limits LD_95_ (lower–upper)	χ2 *P* value
*An. arabiensis* melanic form (M)	1.369 (1.263–1.481)	2.287 (2.053–2.653)	0.742
*An. arabiensis* normal form (N)	0.806 (0.612–0.941)	1.798 (1.547–2.337)	0.778
*An. arabiensis* in polluted habitats	1.682 (1.557–1.805)	2.785 (2.535–3.160)	0.072

P value ≤ 0.05 considered as significant

## Discussion

Until recently, urban development was generally believed to reduce the risk of vector breeding, and thus malaria transmission. However, millions of clinical episodes of malaria occur annually in urban areas, indicating that the epidemiology of this disease is changing [17, 18]. The findings of the present study, together with other studies on other anopheline mosquitoes, indicate a change of *An. arabiensis* breeding requirements in urban settings. This is in line with many studies which stated that *Anopheles* mosquitoes have adapted to new breeding sites created by urbanization [19]. Studies conducted in Accra, Ghana and Dar es Salaam in Tanzania, revealed the presence of *An. gambiae* s.l. in organically polluted water habitats, sewage ponds and in swamps extremely polluted with organic matter [4, 7].

Khartoum State in Central Sudan had a unique Khartoum Malaria-Free Initiative (KMFI), which was launched in 2002 by the State and the Federal Ministry of Health, in collaboration with WHO. The core intervention for KMFI was larval control through weekly application of Temephos and environmental management [20]. The continuous application of Temephos larvicide for more than 13 years might explain the development of resistance to Temephos. *Anopheles arabiensis* from polluted habitats was more tolerant to Temephos compared to *An. arabiensis* from non polluted habitats. This finding is in line with Kabula et al. [2] who suggested that the adaptation of *An. gambiae* s.s. to breed in polluted water may be influenced by their insecticide resistance status and vice versa. It is more likely that resistant gravid female choose the available polluted water as a last chance to lay its eggs when they have no choice during the hot dry season to find an alternative habitat which require more flying during an extreme harsh environmental condition. Aboud et al. [14] reported that the haplotype diversity within melanic populations of *An. arabiensis* was higher than within normal populations and better adapted to hot and arid environments. A high level of genetic diversity is often beneficial to a species as it provides more opportunities for adaptation when a species environment change and might explain the increased tolerance of the melanic form to Temephos insecticide.

The mean level of conductivity reported in this study increased 8.6 times compared to control site. Similar findings was obtained by Tene Fossog et al. [6] in Douala, Cameroon, for conductivity in non-polluted and polluted sites and by Awolola et al. [1] in Lagos, Nigeria. However, in Yaoundé, Cameroon, Antonio-Nkondjio et al. [3] recorded lower values of conductivity in rural and urban habitats. In the present study, the turbidity levels were similar to with the result recorded by Awolola et al. [1]. The simple definition of ‘turbidity’, which favours *Anopheles* larvae in a habitat might not be precise enough. This is because water which is turbid from particles not nutritious for *Anopheles* larvae could disfavour the growth and development of larvae and can be considered a limiting factor [21], while water turbid from food particles represents a very suitable habitat and may be useful for larval development. Some studies showed that *An. arabiensis* and *An. gambiae* s.s. larvae were associated with highly turbid water, with algae and little or no aquatic vegetation, while other studies recorded presence of algae and vegetation as limiting factors [22, 23]. This study reported that all water bodies (permenant or semi permenent) are potential larval habitats and help maintaing the vector populations during the hot dry season. [24] reported that permanence of a habitat had no significant influence on larval productivity and all potential breeding sites need to be considered as source of malaria risk at any time of the year. 

The study confirmed the presence of oil in polluted water but the values was lower compared to the values recorded by Awolola et al. [1] in Lagos, Nigeria, who found that the level of the three heavy metals (Fe, Cu, Pb) was more than twofold higher than those obtained from the control site. However, in this study concentrations of Pb (mg/l) and Cu (mg/l) were less by at least three times in comparison to the control site. The present study found that the mean of nitrate was 4.6 times compared to control site.

The findings of the present study together with other studies from sub-Saharan Africa and other parts of the world, indicates that *An. gambiae* complex is adapting to a wide range of water pollution in urban settings. *Anopheles* larvae are not restricted to clearly defined habitats. Therefore, all water bodies in an urban environment should be considered potential breeding places and must be a target for larval control. Further studies are need to be shown over time, during different seasons, in respect to other positive habitats from the total found in the area to estimate the extent of this adaptation in the targeted area.

## Conclusions

The present study documents the presence of minor populations of *An. arabiensis* larvae in polluted urban habitats in Khartoum state which could have further implications on the biology of malaria vector and the epidemiology of urban malaria in Sudan. The study highlights the need for more precise definitions for larval habitats of malaria vectors.

## Abbreviations

GPS: global positioning system
KMFI: Khartoum malaria free initiative
LC: lethal concentration
LSM: larval source management
mg/l: milligram per litre
ml: millilitre
s.l.: sensu lato
s.s.: sensu stricto
TDS: total dissolved solids
